# Dr. Walker, on Vacciolation

**Published:** 1804-11-01

**Authors:** John Walker


### *To the Editors of the Medical and Physical Journal.*

Gentlemen,

THOUGH not feeling any symptom of the *cacoethes scribendi*, but rather wishing to have done with the occupying of so many of the pages of your valuable Journal, from month to month, I yet find imposed on me the *onus* of taking up the pen. John Ring, the indefatigable champion pion of vacciolation, says, in your last number, (p. 356) that the variolous pustule, excited by inoculation, does not break into a number of pustules. He may be perfectly right in this assertion, yet I cannot help thinking, that in the irregularly formed vesicle running on to a considerable extent under the cuticle, and at length breaking, I have seen the source of those vesicles close by the inoculated part, which, he says, are secondary pustules. Of the pestilential variola, now about to take its departure from the world, what were the local effects when under controul by inoculation? The following statement of them from Woodville's Reports, induces me to think that I have not been quite mistaken in the notion that the variolous pock breaks into a number of distinct vesicles.

"In cases wherein inoculation of the small-pox proves effectual, a small particle of variolous matter being applied by a superficial puncture of the skin, usually produces in the course of three or four days, or sooner, a little elevation of the punctured part, discoverable by the touch, and a red speck distinguishable by the eye. From this time the redness advances in a circular form, more or less rapidly, according to the constitutional circumstances of the patient; and the first effect of this superficial inflammation is the formation of a vesicle upon its centre, which usually appears between the fourth and seventh day after the inoculation. The extent of this vesicle is generally found to bear some proportion to the intensity of the inflammation, and contains a limpid fluid, by the absorption of which, the small-pox is produced. The vesicle soon bursts, and the central part of the puncture becomes depressed, and often of a dark hue; which appearances, together with the marginal inflammation, continue to increase till the eruptive symptoms subside, when the edges of the depressed part begin to swell with a purulent fluid, and the inflammation gradually recedes."

From the importance which I attach to what comes from the pen of a Ring on the subject of vacciola, I am particularly induced to reply to him in defence of the removal of that scab, (p. 243) on the taking of matter, which is sometimes seen in the centre of the pock, from its most incipient state. What he calls a hazardous experiment I consider an act of the most wholesome precaution; I am even strongly persuaded, that most of your readers who practise vacciolation, must have often

 met met with the genuine vesicle, which requires this precaution; and that they must naturally have used it. From p. 303, of your last number, it appears to have met the eye of Jenner. The scab, with "the perfect vaccine fluid in a ring around it," must, I think, have been the pock in question. It is uniformly produced on the application of active matter by such insertion of the lancet as produces rather incision, I mean of cuticle only, than, literally, puncture, though he refers it to another cause.

The ingenious and learned paper of Philologos, (p. 335) renders it unnecessary for me to advance any thing further in defence of vacciola, in opposition to vaccina. Vaccævara appears to me to be the only correct term yet proposed. Exclusively of its being one word, it is preferable to Jenner's variola vaccina, if vara be a more precise term than the diminutive variola for the now departing pestilence.* Till this last correction, (vara for variola) with many others, shall have taken place in medical language, it may be best for the sake of euphony, and because of the similitude even of vacciola to the long adopted term variola, to continue to speak of the vacciolator with vacciolous matter, producing true vacciola in subjects which have never yet had the small-pox. If as great an effect could be produced on the constitution by small-pox inoculation after vacciola as is frequently produced by vacciolation after the small-pox, whether had naturally or by inoculation, the discovery of Jenner would have never made its way in the world as it has happily done. Of medical men and others I know many who, as well as myself, have by design vacciolated themselves after the small-pox. I know some others, both men and women, who have been vacciolated by accident, and who, according to the seat of the wound, have been much affected with cervical, axillar, dorsal, lumbar, or inguinal tumefactions, indurations, or pains.

It happens to me, sometimes frequently in the course of a month, that patients brought to be examined, a week after

* I have little doubt but Philologos could find a short classical compound sufficiently characteristic of the desolating disease. The accommodating inventor of the term vaccine-pock, mingling together Saxon and Latin, might give it such discordant name as *drad-vara*, and might defend the term by saying, our whole language is of heterogeneous mixture; why may not a single term be so? Drad-vara is two or three syllables shorter than *horrida vara*, or *vara horribilis*, and expresses about as much.

after inoculation, present a pock so far advanced, and with so stained and even crustaceous a kind of surface, that I immediately hesitate, and decline taking the matter. On inquiry I find, that the patient has, at different times, been in the way of the small-pox, though he did not know that he had ever had it; or that when an infant, he had a few spots which the relations took for chicken-pox or swine-pox, but which the effect of vacciolation (inoculation of vacciolous matter) shews me at once to have been the small-pox. These different eruptions have often been mistaken for each other. It will be well if those medical gentlemen, numerous and of unquestionable character, who have undertaken to investigate the case in Fullwood's Rents, page 384, Sept. 26, (of which your *status quo, eo tempore,* is very exact) be able to make accurate discrimination. On the seventh evening of the eruption I saw the child, and by candle-light declared it to have very much the appearance of a well marked case of small-pox. A physician present, whose medical report meets the public eye to a very great extent, once a month, said, if it was not a case of small-pox, he had never seen one, and that he should not wonder if it proved fatal. It is not wonderful that he should form such an opinion from the uneasiness of the child. It had been much teazed in its cradle by numerous medical visitants, and many vesicles had been punctured for matter for experiments. He urged the question, whether I did not consider it to be small-pox. Though very much resembling variola, I remarked, it might yet be found to be varicella. On seeing the child running about the next morning, (what kind of small-pox was this?) the eruption on the face in a state of desiccation, the vesicles on its body and limbs plump and full, giving me the same idea of a capability of bearing pressure as a bunch of ripe grapes or currants would, I declared my full conviction of its being varicella. A physician present said the eruption was too watery to be the small-pox. Some surgeons declared themselves more strongly, that it was chicken-pox. A few days afterwards, I accompanied Dr. Woodville to see the case, when, being more advanced, it became proportionally more obscure. He said, on the occasion, that if it should be found to be the small-pox, the cow-pock inoculation stood upon as good ground as the variolous; for that it had happened in the course of a year that several patients had come to the hospital sprinkled with the small-pox, who had previously gone through through the variolous inoculation.* Whether the committee alluded to will be able to satisfy themselves, or only to puzzle themselves the more by the investigation of the effects of the series of inoculation instituted, the commencement appears to have been nearly as legitimate as is possible, in the present state of the question. If the first inoculation produce small-pox, with its most dismal train of subsequent disease, blindness or death, the father of the child will only have to reproach himself, as he had previously sworn he would shoot the practitioner, if, under semblance of inoculating small-pox, he introduced the filthy cow-pock. I hope every member of the committee, before coming to a decision, may peruse the excellent paper of Macdonald, the second article of your last number. In the minds of many, who already most unwaveringly decide, and to whom I must appear ridiculous in venturing yet to doubt, a becoming hesitation may be produced. Hail, Vacciola! Hail! Clearness belongs to thee. Thou hast not a *fac simile.*

John Ring complains, that a certain publication, "ready to receive and circulate any lie that is fabricated against vaccination," refused admission to a respectful representation of the impropriety of admitting anonymous attacks on persons, and anonymous reports on so important a subject as cow-pox. Feeling myself somewhat implicated in the detraction

* Though the declaration of Woodville must alone be sufficient to prevent the vacciolous inoculation from falling into disrepute, if the committee shall at length find that the case under investigation have been a true case of small-pox, yet it may be well for them to remember, that in an institution where so many thousands have annually been inoculated, it is quite a possible case that a mistake might happen in the record; that the vacciolation may have been incomplete. The following piece of egoism will be my sufficient apology with the worthy officers of the *really* original public institution of vacciolation for entertaining the idea. Before the organisation of the Royal Jennerian Society, I, *per se*, I, had gratuitously vacciolated several hundreds in this metropolis. Since my election to their Central House, I have had the happiness of being the instrument of protecting twice as many thousands. The office of Resident Inoculator, in one instance, resembles that of prime minister; he is liable to become a public butt. The attempts to throw me out, did, in the beginning, induce in me such habits of vigilance that I cannot help most confidently trusting, that the "thousands" are as secure from variola as vacciola can render them; of the certain protection of the "hundreds," my hope is not quite so ardent. There may have occurred an instance or two, where I may have taken it for granted that the vacciolation was complete, on proof more equivocal than I should now venture to receive; or I may even in haste have noted cases as complete in the register, from memory, and thus have committed some mistakes.

detraction of my colleague, I offer you some testimonials which are not anonymous; I suppose that the "lie" has grown out of the facts which are stated in the document from Valette.

"To the INHABITANTS of MALTA.

"Some time has now elapsed since Dr. Marshall and Dr. Walker have began to practise the Jennerian inoculation in Valette, and experiments have shewn that no one who has passed through that disease is afterwards capable of taking the small-pox. At the present moment, two children who were inoculated by Dr. Marshall are ill in the small-pox from having, previously to their inoculation with the cow-pox, been exposed to the infection of the small-pox, which, at the time of their being inoculated with the cow-pox, was in the body.

"In one of the children, the part inoculated with the cow-pox matter never inflamed, and the child fell ill with the small-pox in four or five days; in the other, the incised part inflamed, and proceeded as usual till the sixth day, when she also fell ill with the small-pox. These two cases, thus fairly stated, shew that they must have been infected with the small-pox at least three or four days previous to being inoculated with the cow-pox; one of them never inflamed; the other, the moment the small-pox appeared, lost its inflammation and turned to common matter.

"As no doubt many reports, prejudical to the Jennerian inoculation may be spread abroad by interested people respecting these children, it is thought necessary to publish this true statement of the affair, that the inhabitants may be upon their guard against all such reports, and to assure them, that it is impossible for any one that has passed through the true cow-pox ever afterwards to be infected with the small-pox.

Protomedico LUIGI CANEANA,

Il Med. di Palazzo, BR. LOREZO CATTAR."

*Valette, Feb.* 27, 1800.

"General Mem. *Foudroyant, Malta, Dec.* 9, 1800.

"The small-pox having made its appearance on board the Alexander, and other ships in the fleet, the Commander in Chief thinks it necessary to refer the respective Captains to the general memorandums of the 19th October last, and to recommend immediate application to Dr. Marshall and Dr.

Dr. Walker, whose safe and excellent mode of treatment has been experienced on board the Foudroyant, and other ships, in preventing the dreadful effects so often attending the small-pox, which may now so easily be avoided without danger or inconvenience.

"By command of the Vice-Admiral,

"Signed, WILLIAM YOUNG."

"*To the respective Captains, &c.*"

"These are to certify, that Drs. Marshall and Walker have administered the vaccine inoculation to such of the crews of all his Majesty's ships, under my command, at Gibraltar, Minorca, Malta, the Port of Marmorice, and on the coast of Egypt, as had the opportunity, and were desirous of submitting to the operation: That these gentlemen have manifested the greatest assiduity for the extension of the practice, bestowed the most unwearied attention to its successful application, and have, according to the information I have received from all quarters, exhibited it with perfect success.

"Given under my hand, on board his Majesty's ship the Foudroyant, in the Bay of Aboukir, 29th March, 1801.

"KEITH."

"*Camp, four miles from Alexandria, April* 11, 1801.

"This is to certify, that Drs. Marshall and Walker attended at the hospital at Malta, for the purpose of inoculating the respective regiments of the expedition to Egypt, according to the general orders of the late Commander in Chief, Sir Ralph Abercrombie, at which time the small-pox had got into the fleet, and was very fatal.

"Dr. Walker accompanied the expedition, with the approbation of the Commander in Chief, to Egypt, and introduced the new practice into the army in general, which was found effectual in arresting the ravages of the small-pox, those soldiers escaping it who submitted to this operation, and doing their duty as usual, while a few, who neglected the opportunity, were laid up.

"We now experience his services in another way, he having consented to be associated with the surgeon of the brigade of seamen on shore; and from Sir Sydney Smith finding it necessary to have the attendance of the surgeon at a distance from the camp, the medical care of the whole brigade falls upon him.

"Major

"Major General Hutchinson feels a sincere pleasure in recommending Drs. Marshall and Walker (for their indefatigable zeal in the service) to his Royal Highness the Duke of York, who ever takes so lively an interest in whatever renders the situation of the soldier comfortable.

"J. Hely Hutchinson, Major General."

In noticing (Med. and Phys. Journal, Jan. 1803) the remuneration granted us for our services, so honourably testified of by the Lord Keith, I mentioned that the Admiralty had not yet paid off the disbursements they had granted us. To the present first Lord of the Admiralty I have made the representation, and already experienced some generosity from himself. I hope shortly to be able to add, that he has obtained us payment from the board.

Yours, respectfully,

JOHN WALKER.

